# Bile duct injury with formation of right hepatic duct-duodenal fistula after cholecystectomy: A case report

**DOI:** 10.1097/MD.0000000000036565

**Published:** 2023-12-08

**Authors:** Yuxu Wang, Yanyan Liu, Pan Lv, Hao Li, Weiqiang Gong

**Affiliations:** a Weifang People’s Hospital, Hepatobiliary and Pancreatic Medicine Center, Weifang, Shandong, China.

**Keywords:** bile duct injuries, cholecystectomy, surgical repair

## Abstract

**Rationale:**

The management of bile duct injury (BDI) remains a considerable challenge in the department of hepatobiliary and pancreatic surgery. BDI is mainly iatrogenic and mostly occurs in laparoscopic cholecystectomy (LC). After more than 2 decades of development, with the increase in experience and technological advances in LC, the complications associated with the procedure have decreased annually. However, bile duct injuries (BDI) still have a certain incidence, the severity of BDI is higher, and the form of BDI is more complex.

**Patient concerns:**

We report the case of a patient who presented with bile duct injury and formation of a right hepatic duct-duodenal fistula after LC.

**Diagnoses:**

Based on the diagnosis, a dissection was performed to relieve bile duct obstruction, suture the duodenal fistula, and anastomose the right and left hepatic ducts to the jejunum.

**Intervention:**

Based on the diagnosis, a dissection was performed to relieve bile duct obstruction, suture the duodenal fistula, and anastomose the right and left hepatic ducts to the jejunum.

**Outcomes:**

Postoperative recovery was uneventful, with normal liver function and no complications, such as anastomotic fistula or biliary tract infection. The patient was hospitalized for 11 days postoperatively and discharged.

**Lessons:**

The successful diagnosis and treatment of this case and the summarization of the imaging features and diagnosis of postoperative BDI have improved the diagnostic understanding of postoperative BDI and provided clinicians with a particular clinical experience and basis for treating such diseases.

## 1. Introduction

Globally, laparoscopic cholecystectomy (LC) remains the preferred modality for treating gallstones, gallbladder polyps, adenomyosis, and other related diseases.^[1,2]^ After more than 2 decades of development, the complications associated with the procedure have decreased yearly with increased experience and technological advances in LC.^[3]^ However, bile duct injuries (BDI) have not significantly reduced and still have a particular incidence, and even the type and severity of BDI have become more complex.^[4]^ There are many causes of BDI that can be observed in all types of biliary surgery, primary gastric resection, and partial hepatectomy.^[5,6]^ Cholecystectomy is the most common procedure, but the incidence of associated BDI is 2 to 3 times higher than that of open cholecystectomy.^[7]^ The consequences of some types of BDI are severe, requiring partial hepatectomy or liver transplantation, and in some cases can even lead to death.^[8]^ The condition often worsens if BDI are not adequately diagnosed or promptly treated.^[9]^ Therefore, a standardized surgical operation is crucial for reducing bile duct injury.^[10]^ For patients who cannot determine whether there is a bile duct injury and the type of bile duct injury, strict preoperative examinations are needed, such as intensive MRI and duodenoscopy + retrograde cholangiopancreatography, which can not only accurately determine the location of the patient bile duct injury and the presence of obstruction and biliary fistulae, but also help determine the indications for cesarean dissection surgery accurately.^[11]^ We report a case of complex BDI after LC in which the common bile duct was clamped, the common hepatic duct was absent, the left hepatic duct was obstructed, and a fistula formed between the right hepatic duct and the duodenum. We also summarize the imaging characteristics and diagnosis of postoperative BDI in combination with the literature to improve the diagnostic understanding of postoperative bile duct injury.

## 2. Case report

**Figure 1.**
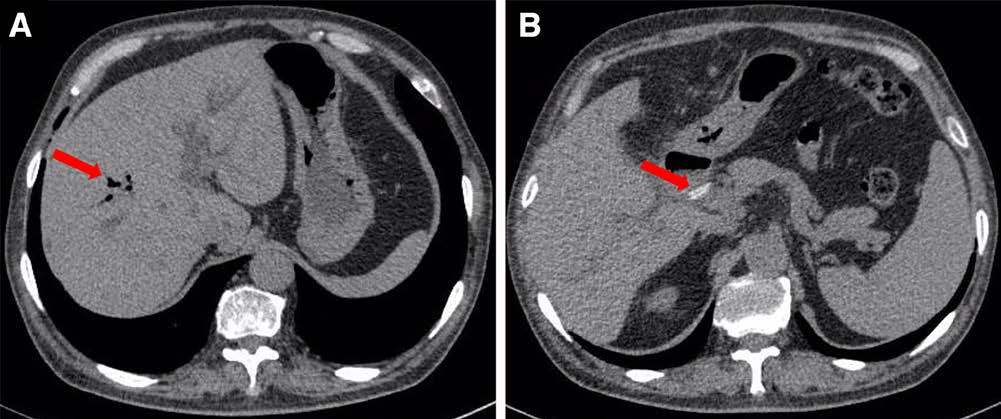
A and B are preoperative CT scans of the upper abdomen; the red arrows in A and B represent gas and hemlock clamps, respectively. CT = computed tomography.

**Figure 2.**
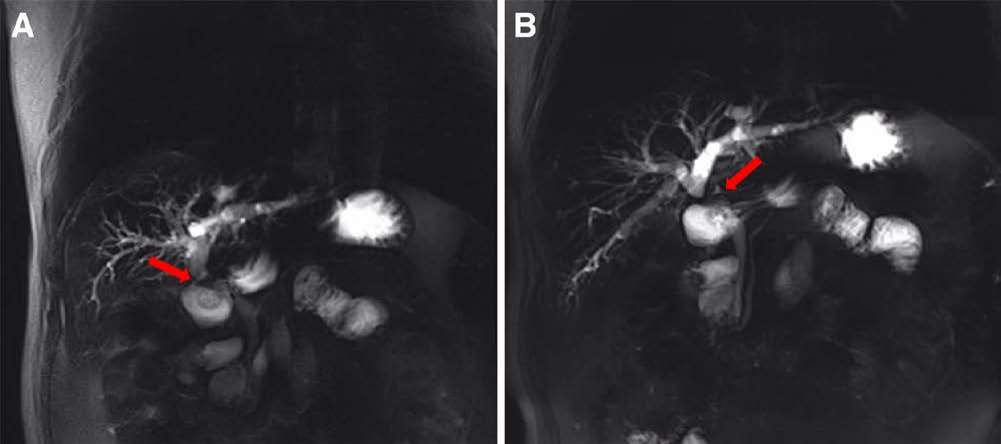
A and B are preoperative MR + MRCP scans of the upper abdomen; the red arrows in A and B represent the formation of a biliary fistula between the right hepatic duct and the duodenum and the absence of the common hepatic duct, respectively.

A 65-year-old man, with recurrent right upper abdominal pain for over half a year, was found to have gallstones in the gallbladder and common bile duct on an enhanced computed tomography (CT) scan of the upper abdomen. According to the *Practice Guidelines for the Diagnosis and Treatment of Biliary Tract Stones* published by the Biliary Surgery Group of the Chinese Medical Association Surgical Branch, the patient indications for surgery were clear, and the relevant presurgical examinations ruled out contraindications to surgery. LC and endoscopic duodenal papillary muscle incision for stone extraction were performed in May 2021, which procedures are widely available in primary hospitals in China. After the surgery, the abdominal drain drained a large amount of bile-like fluid, thought to be a fistula in the stump of the cystic duct, which was removed after 2 months of continuous abdominal drainage because of the decrease in drainage flow. The patient had recurrent chills and high fever for 5 months, and his symptoms improved after anti-infective treatment at a local hospital. Two days prior, he had chills and high fever again, with a temperature of 39.8ºC. An enhanced CT scan of the whole abdomen showed intrahepatic bile duct dilatation and the presence of gas. For further diagnosis and treatment, the patient visited our hospital (Weifang People Hospital) for consultation. Test results after admission:

• Leukocytes 12.58*10^9^/L, percentage of neutrophils 91.5%, percentage of lymphocytes 18%, erythrocytes 3.08*10^12^/L, platelets 314*10^9^/L, rapid C-reactive protein 37.1 mg/L, calcitoninogen 0.648 µg/L.
• Albumin transaminase 65 U/L, glutamine transaminase 49 U/L, alkaline phosphatase 445 U/L, glutamyl transpeptidase 960 U/L, total bilirubin 66.8 µmol/L, direct bilirubin 51.6 µmol/L, indirect bilirubin 15.2 µmol/L, albumin 31.4 g/L, blood amylase 48 U/L.
• CT scan and enhancement of the upper abdomen showed dilatation of the intrahepatic bile ducts with pneumatosis, and hyperdense shadows were observed in the hepatoportal region (Fig. 1).
• MR scan and MRCP of the upper abdomen showed apparent dilatation and pneumatization of the intrahepatic bile ducts and the right and left hepatic ducts; the common hepatic duct was not visualized; the common bile duct was well traveled and did not show apparent dilatation; and the gallbladder was not visible (Fig. 2).

**Figure 3.**
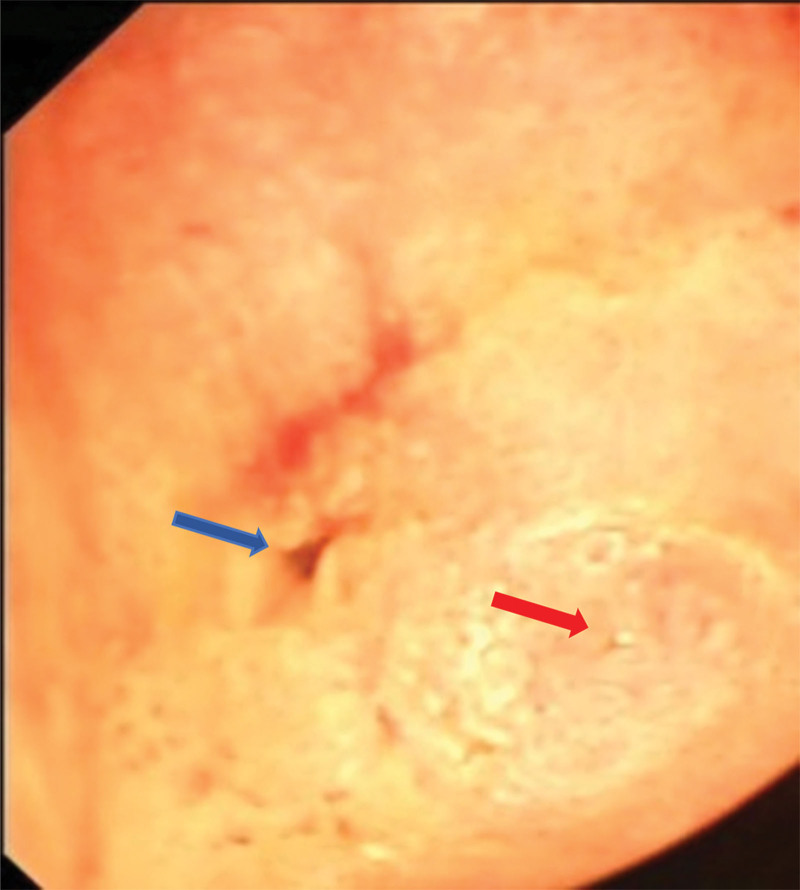
Duodenoscopic view of the fistula formed by the right hepatic duct and duodenum as indicated by the blue arrow, and the biliopancreatic pot-belly at the opening of the duodenum as indicated by the red arrow.

Based on the patient medical history, bile duct injury, bile duct stenosis, and biliary tract infection after cholecystectomy were considered. In addition to giving the necessary symptomatic treatment, it was decided to perform transendoscopic retrograde cholangiopancreatography for examination, and the results of the test: the esophagus and stomach did not show any obvious abnormality, the duodenal papilla was found, and after intubation and entering the cholangiogram, the common bile duct was seen to be interrupted, and because of the large amount of bile in the duodenum, repeated intubation was done to search for the source of bile, and bile was seen to be outflowed from the opening of the intestinal wall of the bulb of the duodenum, the right lobe of the liver was seen to be revealed by imaging The incision knife could not be inserted and further operation was abandoned (Fig. 3). Based on the findings of transendoscopic retrograde cholangiopancreatography combined with an MR scan of the upper abdomen, it was considered that the common bile duct had been incorrectly clamped during the operation for cholecystectomy, the common hepatic duct was defective, the left hepatic duct was adherent to the surrounding tissues to form an obstruction, and the right hepatic duct created a biliary fistula with the duodenum. The decision was made to perform a cesarean section, and appropriate management was provided according to the conditions encountered during the operation. During the surgery, dense adhesions between the stomach and duodenum were observed in the porta hepatis, and a hard hemo-lock clip was detected in the porta hepatis. The adhesions in the porta hepatis were separated, and the extrahepatic bile ducts were carefully freed. A fistula was observed between the right hepatic duct and the duodenal bulb, with a transverse section of the bile duct in the porta hepatis, the common hepatic duct, and the superior end of the common bile duct missing. The lower end of the common bile duct was dissected and closed with 2 hemo-lock clips. After removal of the distal hemo-lock clamp of the common bile duct, the duodenum was explored and the distal bile duct was sutured. The right hepatic duct was separated from the duodenal fistula and the duodenal fistula was sutured. The first hepatic hilum was revealed, and a large amount of bile flowed from the right and left hepatic ducts, which were anastomosed to the jejunum. The patient recovered smoothly without obvious postoperative complications and was discharged 11 days after the operation. The patients were reexamined every 3 months after the operation for 2 years, and no significant complications was found.

## 3. Discussion

The management of BDI remains a considerable challenge in hepatobiliary surgery. Biliary surgeons worldwide have been working on preventing and treating BDI. BDI not only lead to extremely serious complications such as biliary fistulae, jaundice, and bile duct stenosis, which affect the long-term prognosis of the patients, but also increase the unnecessary medical burden.^[12]^ BDI occur mainly during cholecystectomy.^[13]^ The incidence of BDI in open cholecystectomy is 0.125% to 0.3%,^[14]^ whereas LC has a high incidence of BDI of 0.4% to 0.6%,^[15]^ and the complexity of the injury is increased. With advancements in surgical techniques, single-incision LC and robot-assisted cholecystectomy are available, but the incidence of BDI has also been reported.^[16]^ In this case, the patient underwent LC, endoscopic duodenal papillomastoidotomy, and lithotripsy in a primary care hospital for choledocholithiasis and gallbladder stones with acute or chronic cholecystitis. Because of the severe inflammation of the gallbladder, important anatomical structures such as the cystic duct, cystic artery, common bile duct, and even the right hepatic artery were poorly visualized. Because of the lack of surgical experience in primary care hospitals, the common hepatic duct was excised as if it were the cystic duct when the gallbladder was resected, and the common bile duct was incorrectly clamped. Therefore, the prevention of BDI may require more rigorous training in biliary surgery and clear identification of the structures in the Calot triangle.

Improving the success rate of bile duct injury repair and reducing the recurrence of biliary fistulae and restenosis after repair are currently considerable challenges in hepatobiliary surgery. It has been well documented that the success of the final repair of BDI depends on the accurate preoperative assessment of the type of injury and selection of the appropriate surgical approach to repair.^[17]^ The surgeon should accurately assess all details of the bile duct injury before performing definitive repair surgery. This mainly includes the site of bile duct injury or stenosis, presence or absence of injury to the right and left hepatic ducts, presence or absence of combined vascular injury, and presence or absence of secondary bile leakage, infection, sclerosing cholangitis, and biliary cirrhosis. Based on a comprehensive evaluation of the above detailed information, the type of bile duct injury can be determined, which is the basis for selecting an appropriate surgical approach to ensure the success of repair surgery.^[18]^ In this case, preoperative MR + MRCP of the upper abdomen showed significant dilatation and pneumatization of the intrahepatic bile ducts and the right and left hepatic ducts. The common hepatic duct was not visualized, which indicated that the patient had missing the common hepatic duct, and the right and left hepatic ducts were impaired. Endoscopic retrograde cholangiopancreatography revealed that the bile ducts duodenally formed biliary fistulas. Therefore, in complicated cases, hepatobiliary surgeons should obtain complete images of the bile ducts to understand the details of the bile duct injury before the final repair of the bile duct injury.

The patient recurrent preoperative high fever and chills, combined with the history, were considered to have developed an infection of the biliary system. Repair or digestive tract reconstruction surgery should be performed without localized or abdominal infection.^[19]^ Based on this principle, we provided the patient with sufficient tertiary cephalosporins for preoperative anti-infective treatment. One-stage repair can be performed for BDI detected soon after surgery if no biliary infection occurs.^[20]^ Although early opinions suggested that delayed repair should be performed at least 3 months after injury, current evidence indicates that final repair surgery can be performed 4 to 6 weeks after effectively controlling local inflammation and infection.^[21]^ In our case, according to the patient bile duct injury, we sutured the distal common bile duct, anastomosed the right and left hepatic ducts to the jejunum, and finally sutured the fistula in the duodenum, and because of the absence of the common hepatic duct we gave up on the choledochal-bile duct anastomosis,^[22]^ so the clinician should analyze according to the type of the mono-ductal injury, the duration of the biliary obstruction, the degree of hepatic damage, and the patient general condition to determine the optimal repair surgical approach, and many repair surgeries fail because the above basic principles are not followed. Any repair and reconstruction surgery should use fine anastomotic techniques to restore the integrity of the bile duct and its continuity with the intestine, and interrupted or continuous mucosal-mucosal anastomoses should be selected with noninvasive suture needles. The anastomosis should be tight to prevent postoperative bile leakage, and care should be taken to avoid over-tightening to impair the blood supply to the anastomotic tissue. The primary purpose of biliary drainage after the final biliary repair is not to maintain the anastomosis but to provide postoperative biliary decompression to prevent bile leakage and access for subsequent angiography or cholangioscopy.^[23]^ Therefore, routine biliary drainage is unnecessary. Short-term drainage may be performed only in cases of unsatisfactory anastomosis, significant inflammation of the bile duct wall, or intrahepatic bile duct stones; the drainage duration usually does not exceed 12 weeks.

In conclusion, management of bile duct injury after LC remains a considerable challenge in hepatobiliary surgery. The successful management in this case was attributed to a series of perfect preoperative examinations, the choice of surgical modality for repair, and precise surgical techniques, which provides clinicians with some clinical experience in diagnosing and treating such diseases, which this provides clinicians with some clinical experience in diagnosing and treating such diseases. Besides, extensive surgical experience is of paramount importance for the prevention of BDI. It is believed that the most beneficial treatment for patients with bile duct injury can be determined by further research in the future.

## Acknowledgments

The authors thank doctors, nurses, and clinical staff who provided care for the patient.

## Author contributions

**Conceptualization:** Hao Li, Yanyan Liu.

**Funding acquisition:** Hao Li.

**Writing – original draft:** Hao Li, Yuxu Wang, Pan Lv, Weiqiang Gong.

**Writing – review & editing:** Yuxu Wang, Pan Lv, Weiqiang Gong.

## References

[1] Cheslyn-Curtis S Emberton M Ahmed H. Bile duct injury following laparoscopic cholecystectomy. Br J Surg. 1992;79:231–2. 1532527 10.1002/bjs.1800790314

[2] Attili AF Carulli N Roda E. Epidemiology of gallstone disease in Italy: prevalence data of the Multicenter Italian Study on Cholelithiasis (MICOL). Am J Epidemiol. 1995;141:158–65. 7817971 10.1093/oxfordjournals.aje.a117403

[3] Lau WY Lai EC Lau SH. Management of bile duct injury after laparoscopic cholecystectomy: a review. ANZ J Surg. 2010;80:75–81. 20575884 10.1111/j.1445-2197.2009.05205.x

[4] Hogan NM Dorcaratto D Hogan AM. Iatrogenic common bile duct injuries: increasing complexity in the laparoscopic era: a prospective cohort study. Int J Surg. 2016;33 Pt A:151–6. 27512909 10.1016/j.ijsu.2016.08.004

[5] Wang Z Wang M Duan F. Bile duct injury after transcatheter arterial chemoembolization: risk factors and clinical implications. Hepatogastroenterology. 2014;61:947–53. 26158147

[6] Westerkamp AC Mahboub P Meyer SL. End-ischemic machine perfusion reduces bile duct injury in donation after circulatory death rat donor livers independent of the machine perfusion temperature. Liver Transpl. 2015;21:1300–11. 26097213 10.1002/lt.24200

[7] Wysocki AP. Population-based studies should not be used to justify a policy of routine cholangiography to prevent major bile duct injury during laparoscopic cholecystectomy. World J Surg. 2017;41:82–9. 27468742 10.1007/s00268-016-3665-0

[8] Hariharan D Psaltis E Scholefield JH. Quality of life and medico-legal implications following iatrogenic bile duct injuries. World J Surg. 2017;41:90–9. 27481349 10.1007/s00268-016-3677-9

[9] Maddah G Rajabi Mashhadi MT Parvizi Mashhadi M. Iatrogenic injuries of the extrahepatic biliary system. J Surg Res. 2017;213:215–21. 28601317 10.1016/j.jss.2015.11.032

[10] Duncan CB Riall TS. Evidence-based current surgical practice: calculous gallbladder disease. J Gastrointest Surg. 2012;16:2011–25. 22986769 10.1007/s11605-012-2024-1 PMC3496004

[11] Williamson JM. Traumatic injuries to the biliary tree. Br J Hosp Med (Lond). 2013;74:138–43. 23665782 10.12968/hmed.2013.74.3.138

[12] Mayo WJ. VI. Some remarks on cases involving operative loss of continuity of the common bile duct: with the report of a case of anastomosis between the hepatic duct and the duodenum. Ann Surg. 1905;42:90–6. 17861664 10.1097/00000658-190507000-00006 PMC1425944

[13] Malla BR Rajbhandari N Karmacharya RM. Management of bile duct injury following cholecystectomy. J Nepal Health Res Counc. 2020;18:214–8. 32969380 10.33314/jnhrc.v18i2.1579

[14] Martin D Uldry E Demartines N. Bile duct injuries after laparoscopic cholecystectomy: 11-year experience in a tertiary center. Biosci Trends 2016;10:197–201. 27319974 10.5582/bst.2016.01065

[15] Waage A Nilsson M. Iatrogenic bile duct injury: a population-based study of 152 776 cholecystectomies in the Swedish Inpatient Registry. Arch Surg. 2006;141:1207–13. 17178963 10.1001/archsurg.141.12.1207

[16] Joseph M Phillips MR Farrell TM. Single incision laparoscopic cholecystectomy is associated with a higher bile duct injury rate: a review and a word of caution. Ann Surg. 2012;256:1–6. 22664556 10.1097/SLA.0b013e3182583fde

[17] Törnqvist B Strömberg C Akre O. Selective intraoperative cholangiography and risk of bile duct injury during cholecystectomy. Br J Surg. 2015;102:952–8. 25919401 10.1002/bjs.9832

[18] Fidelman N Kerlan RK Jr. Laberge JM. Accuracy of percutaneous transhepatic cholangiography in predicting the location and nature of major bile duct injuries. J Vasc Interv Radiol. 2011;22:884–92. 21514840 10.1016/j.jvir.2011.02.007

[19] Sulpice L Garnier S Rayar M. Biliary cirrhosis and sepsis are two risk factors of failure after surgical repair of major bile duct injury post-laparoscopic cholecystectomy. Langenbecks Arch Surg. 2014;399:601–8. 24796956 10.1007/s00423-014-1205-7

[20] Kirks RC Barnes TE Lorimer PD. Comparing early and delayed repair of common bile duct injury to identify clinical drivers of outcome and morbidity. HPB (Oxford). 2016;18:718–25. 27593588 10.1016/j.hpb.2016.06.016 PMC5011094

[21] de Reuver PR Grossmann I Busch OR. Referral pattern and timing of repair are risk factors for complications after reconstructive surgery for bile duct injury. Ann Surg. 2007;245:763–70. 17457169 10.1097/01.sla.0000252442.91839.44 PMC1877064

[22] Halbert C Altieri MS Yang J. Long-term outcomes of patients with common bile duct injury following laparoscopic cholecystectomy. Surg Endosc. 2016;30:4294–9. 26823055 10.1007/s00464-016-4745-9

[23] Murr MM Gigot JF Nagorney DM. Long-term results of biliary reconstruction after laparoscopic bile duct injuries. Arch Surg. 1999;134:604–9; discussion 609. 10367868 10.1001/archsurg.134.6.604

